# Treatment of dementia and mild cognitive impairment with or without cerebrovascular disease: Expert consensus on the use of *Ginkgo biloba* extract, EGb 761^®^

**DOI:** 10.1111/cns.13095

**Published:** 2019-01-15

**Authors:** Nagaendran Kandiah, Paulus Anam Ong, Turana Yuda, Li‐Ling Ng, Kaysar Mamun, Reshma Aziz Merchant, Christopher Chen, Jacqueline Dominguez, Simeon Marasigan, Encarnita Ampil, Van Thong Nguyen, Suraya Yusoff, Yee Fai Chan, Fee Mann Yong, Orapitchaya Krairit, Chuthamanee Suthisisang, Vorapun Senanarong, Yong Ji, Ramesh Thukral, Ralf Ihl

**Affiliations:** ^1^ Duke‐NUS Singapore National Neuroscience Institute Singapore Singapore; ^2^ Hasan Sadikin General Hospital Bandung Indonesia; ^3^ School of Medicine and Health Science Atma Jaya Catholic University of Indonesia Jakarta Indonesia; ^4^ Changi General Hospital Singapore Singapore; ^5^ Singapore General Hospital Singapore Singapore; ^6^ Department of Medicine National University of Singapore Singapore Singapore; ^7^ Department of Pharmacology National University of Singapore Singapore Singapore; ^8^ St Luke's Medical Center Quezon City Philippines; ^9^ University of Santo Tomas Metro Manila Philippines; ^10^ Can Tho University of Medicine and Pharmacy Can Tho Vietnam; ^11^ Hospital Sultan Ismail Johor Malaysia; ^12^ Hospital Kuala Lumpur Kuala Lumpur Malaysia; ^13^ Subang Jaya Medical Centre Selangor Malaysia; ^14^ Ramathibodi Hospital Bangkok Thailand; ^15^ Faculty of Pharmacy Mahidol University Bangkok Thailand; ^16^ Siriraj Hospital Bangkok Thailand; ^17^ Beijing Tiantan Hospital Capital Medical University Beijing China; ^18^ Sahara Hospital Lucknow India; ^19^ Alexian Hospital Krefeld Germany

**Keywords:** Alzheimer disease, cerebrovascular disease, dementia, EGb 761^®^, *Ginkgo biloba*

## Abstract

**Background:**

The *Ginkgo biloba* special extract, EGb 761^®^ has been widely used in the treatment of neuropsychiatric disorders, including Alzheimer’s disease (AD).

**Methods:**

To guide clinical practice in the Asian region, the Asian Clinical Expert Group on Neurocognitive Disorders compiled evidence‐based consensus recommendations regarding the use of EGb 761^®^ in neurocognitive disorders with/without cerebrovascular disease.

**Results:**

Key randomized trials and robust meta‐analyses have demonstrated significant improvement in cognitive function, neuropsychiatric symptoms, activities of daily living (ADL) and quality of life with EGb 761^®^versus placebo in patients with mild‐to‐moderate dementia. In those with mild cognitive impairment (MCI), EGb 761^®^ has also demonstrated significant symptomatic improvement versus placebo. World Federation of Societies of Biological Psychiatry guidelines list EGb 761^®^ with the same strength of evidence as acetylcholinesterase inhibitors and N‐methyl‐D‐aspartate (NMDA) antagonists e.g. memantine (Grade 3 recommendation; Level B evidence). Only EGb 761^®^ had Level B evidence in improving cognition, behaviour, and ADL in both AD and vascular dementia patients. Safety analyses show EGb 761^®^ to have a positive risk‐benefit profile. While concerns have been raised regarding a possible increased bleeding risk, several randomized trials and two meta‐analyses have not supported this association.

**Conclusions:**

The Expert Group foresee an important role for EGb 761^®^, used alone or as an add‐on therapy, in the treatment of MCI and dementias, particularly when patients do not derive benefit from acetylcholinesterase inhibitors or NMDA antagonists. EGb 761^®^ should be used in alignment with local clinical practice guidelines.

## 1 INTRODUCTION

The rapid aging of the global population is resulting in an increasing prevalence of mild cognitive impairment (MCI) and dementias.^1^, ^2^ Age is a key risk factor for dementia and Alzheimer's disease (AD)^1^; after the age of 65 years, the prevalence of dementia doubles with every five additional years.^2^ Prevalence is higher among women than men, largely because women tend to live longer.^2^

In 2018, the number of individuals worldwide with dementia was estimated to be 50 million. This number has been projected to increase exponentially to 82 million by 2030, and to 152 million by 2050.^4^ In high‐income countries, estimates of dementia prevalence are around 5%‐10% in the population aged 65 years or older^2^; however, evidence suggests the prevalence in such countries may be reaching a plateau.^5^, ^6^ In the developing world, however, the increasingly maturing population is expected to be mirrored by a continued rise in the prevalence of dementia,^3^, ^7^, ^8^ with an estimated 68% of dementia cases expected to be found in low‐to‐middle income countries by the year 2050.^3^

Asian data indicate a crude estimated dementia prevalence of 4.7% in the over‐60 population; although there is country‐to‐country variation.^3^, ^8^ National reports of dementia prevalence in various Asian countries range between approximately 2%‐13%,^8^, ^9^ and this is expected to rise dramatically in coming years.^8^

Cerebrovascular disease (CVD) frequently coexists with AD. In Asia, AD + CVD accounts for up to 20% of all dementia cases,^9^ and there is some evidence for a higher prevalence of AD + CVD in the Asian region compared with Western populations.^10^ Globally, but also in Asia, AD + CVD is thought to be underdiagnosed, primarily due to lack of awareness resulting from a lack of defined diagnostic criteria, but also due to the high cost of investigations, a lack of adequate diagnostic facilities, and other resource constraints.^9^ It has been suggested that some Asian epidemiology studies may be influenced by “survivor bias” due to a high proportion of early deaths.^9^ Epidemiology study methodologies and findings are also heterogeneous; among dementia patients across Asia, estimated proportions of dementia patients with AD + CVD range between 7% (Sri Lanka) and 50% (Japan).^9^

Dementias place an appreciable burden on patients, their families, and the healthcare system. Quality of life (QoL) is decreased for patients as well as for their families and caregivers, and a severe cost burden also results from long‐term medication use, hospitalization, and home‐based healthcare.^2^, ^11^

### 1.1 Definitions and symptoms

Mild cognitive impairment is understood to be a neurocognitive state between normal cognitive aging and dementia, and can be an early manifestation of, and a risk factor for, AD or other neurodegenerative disorders.^12^, ^13^ MCI is not linked to any specific etiology,^13^ but in the context of AD biomarkers, MCI is considered to be a prodromal stage of AD dementia.^12^ MCI is characterized by mild impairment in one or more cognitive domains, but, in contrast to overt AD dementia, no significant social or occupational impairments are present; individuals are generally able to maintain independence in daily functioning with minimal assistance.^12^, ^14^, ^15^

Clinically overt dementia has a spectrum ranging from mild to severe.^15^ Diagnosis involves clinical assessment, neuropsychological evaluation, and often neuroimaging.^9^ Core clinical criteria for all‐cause dementia, based on DSM‐5 criteria for neurocognitive disorders, include cognitive impairments and behavioral symptoms that interfere with functioning and activities of daily living (ADL). Typical cognitive symptoms may include impairments in complex attention, executive function, learning and memory, language function, perceptual motor function, and social cognition.^15^ Noncognitive behavioral and psychological symptoms of dementia (BPSD), also known as neuropsychiatric symptoms (NPS), are observed across the severity spectrum of dementia,^16^ and can include changes in personality or usual conduct, anxiety, agitation, elation, irritability, apathy/indifference, depression/dysphoria, disinhibition, aberrant motor behavior, sleep or appetite changes, and hallucinations or delusions.^15^, ^16^

The term “AD dementia” specifically refers to a clinical syndrome arising secondary to the pathophysiological process that leads to AD.^15^ AD and vascular dementia (VaD) are considered to be the most common forms of dementia,^9^ but more recently, attention has focused on the co‐existence of AD and CVD.^18^, ^19^ AD and VaD may be considered to be a disease continuum, with pure AD at one end of the spectrum, VaD at the other, and AD with concomitant CVD comprising the majority of cases.^18^, ^21^, ^22^ AD and CVD contribute independently, but additively, to the risk of dementia.^23^

It is important to distinguish between AD, VaD, and AD + CVD, because of differences in clinical course and cognitive profiles, and differential treatment responses.^9^ Diagnosis of AD + CVD can be made when (a) clinical signs of AD dementia are accompanied by evidence of CVD (vascular lesions) on brain imaging; or (b) dementia clinically presenting as sudden‐onset, is accompanied by biomarker evidence of AD such as amyloid plaques on positron emission tomography.^9^ The neuroimaging hallmarks of CVD include chronic lacunes, white matter hyperintensities (WMH), perivascular spaces, and microbleeds.^9^ CVD has been demonstrated to be a risk factor for progression from MCI to AD.^24^ WMH have also been shown to be independently associated with BPSD.^25^

### 1.2 Management options

Goals of dementia treatment are stabilization or slowing of disease progression, reduction in psychological and behavioral symptoms, improvement of QoL, and reduction of caregiver burden.^9^

Acetylcholinesterase inhibitors (AChEIs; eg, donepezil, rivastigmine, and galantamine) are first‐line agents for the treatment of AD and AD + CVD in Asia. Second‐line options include N‐methyl‐D‐aspartate (NMDA) receptor antagonists (eg, memantine).^9^

The *Ginkgo biloba* special extract, EGb 761^®^ has also been used widely in the treatment of cognitive disorders, including AD and AD + CVD.^26^, ^27^ Different parts of the *Ginkgo biloba* tree have been used for centuries in Chinese herbal medicine to treat a variety of health conditions, and evidence for the use of EGb 761^®^ in the symptomatic treatment of dementia, particularly in the presence of NPSs, dates back more than two decades.^27^, ^28^ The EGb 761^®^ extract is derived through a specific, proprietary process under strict procedural control. EGb 761^®^ is a dry extract from *Ginkgo biloba* leaves, adjusted to 22.0%‐27.0% ginkgo flavonoids and 5.0%‐7.0% terpene lactones consisting of 2.8%‐3.4% ginkgolides A, B, and C, and 2.6%‐3.2% bilobalide, and containing less than 5 ppm ginkgolic acids. Depending on local regulatory frameworks, EGb 761^®^ is classified as a drug in some countries, including China, the Philippines, Thailand and Vietnam. In Singapore and Malaysia, EGb 761^®^ is currently classified as a supplement; in Indonesia, it is classified as a “phytopharmaceutical.”

EGb 761^®^ exhibits various beneficial properties in patients with AD and AD + CVD. The molecular basis for these effects is not fully understood; however, the extract appears to have neuroprotective properties. It is a polyvalent free radical scavenger that improves mitochondrial function, decreases blood viscosity and enhances microperfusion, modulates serotonin levels in various brain areas, increases dopamine levels in prefrontal cortical areas, decreases amyloid‐β fibrillogenesis, and attenuates a hyperactivated hypothalamus‐pituitary‐adrenal axis.^32^, ^33^ In a study by Rodríguez et al in 2007, EGb 761^®^ at the recommended dose of 240 mg daily (in single or divided doses) was shown to effectively diminish arteriosclerotic nanoplaque formation and size in patients with coronary artery disease. In the long term, this is thought to result in beneficial effects on cerebral blood flow.^41^

Herein, we summarize evidence‐based consensus recommendations of an Asian Clinical Expert Panel regarding the use of EGb 761^®^ in neurocognitive disorders with or without CVD. The purpose of these recommendations is to help guide clinical practice in the Asia region, with the aim of improving the care of patients with MCI and dementias.

## 2 METHODS

The Asian Clinical Expert Group on Neurocognitive Disorders consists of twenty members including neurologists, geriatricians, psychiatrists and a pharmacist, from around the Asia region. There was also involvement of an expert advisory member from Germany.

The group convened in 2017 to critically review the published literature on *Ginkgo biloba* extract EGb 761^®^ in the treatment of AD, VaD and BPSD, and worked together to develop a series of consensus statements on the role of EGb 761^®^ in the treatment of these conditions. All members were provided prior with all relevant English language publications on the use of EGb 761^®^ in dementia and MCI with or without CVD, and were given sufficient time to review the literature. Experts then completed a pre‐meeting survey before gathering to discuss amendments to a series of proposed consensus statements. On the day of the consensus meeting, two presentations on key published findings were delivered by experts in the field. All proposed statements were then discussed in depth, and when necessary, polling of Expert Group members was carried out to choose or formulate statements that were accepted unanimously or by majority vote.

This document is intended as a collection of consensus recommendations only, and is not designed to be used as a treatment algorithm. Experts’ clinical opinions were based on the available data, and were unrelated to different regulatory classifications in respective countries.

## 3 RESULTS

The Expert Group identified English language articles relevant to the use of EGb 761^®^ in MCI, AD, VaD, and BPSD in the literature. The Group also identified a number of salient aspects relating to the use of EGb 761^®^ in clinical practice, and formed specific consensus recommendations based on the available evidence (Table 1). Key trials evaluating EGb 761^®^ are summarized in Table 2, and key meta‐analyses are discussed below.

**Table 1:** Summary of expert consensus statements from the Asian Clinical Expert Group on Neurocognitive Disorders

*Ginkgo biloba* extract EGb 761^®^ has a role in the management of AD, VaD, BPSD, and MCI	
**Consensus statement 1a: Efficacy of EGb 761^®^ in AD, VaD, and BPSD**	
Based on the available evidence, the Expert Group consider current best practice for the pharmacological treatment of AD (±CVD), VaD, and BPSD to be as follows (best practice may vary between countries): AD: AChEI, memantine, *Ginkgo biloba *(EGb 761^®^) VaD: AChEI, memantine, *Ginkgo biloba *(EGb 761^®^), antiplatelet therapy BPSD: ChEI, nonpharmacological treatment, antipsychotics (off‐label), memantine, SSRIs, sedatives, and EGb 761^®^	Expert recommendation For EGB 761^®^ specifically: Class of recommendation I; level of evidence A
**Consensus statement 1b: Management of MCI**	
EGb 761^®^ may be considered for use in patients with MCI	Class of recommendation IIB; level of evidence A
**Consensus statement 1c: How to use EGb 761^®^**	
EGb 761^®^ can be used as a single agent where deemed appropriate in certain individuals. It is important to allow sufficient time for the effects of EGb 761^®^ to become evident	Class of recommendation I; level of evidence A
EGb 761^®^ can be used as an add‐on agent, in combination with standard anti‐dementia drugs	Class of recommendation IIb; level of evidence B
**Consensus statement 1d: EGb 761^®^ dosage**	
Given the clinical data available to date, EGb 761^®^ at daily dose of 240 mg is an evidence‐based treatment option for the management of AD, VaD, and mixed dementia (AD + CVD) Evidence suggests that EGb 761^®^ 240 mg/d has efficacy comparable with AChEIs and memantine in the treatment of AD, including improvements in cognition, BPSD, and daily function in VaD and mixed dementia (AD + CVD)	Class of recommendation IIA; level of evidence A
**Consensus statement 1e: Lack of efficacy or intolerance of standard drugs may warrant use of EGb 761^®^**	
The use of EGb 761^®^ for the treatment of AD, VaD, and mixed dementia (AD with CVD) is particularly warranted when patients are unable to tolerate the side effects of cholinesterase inhibitors or memantine, or there is a lack of efficacy with standard treatments	Expert recommendation

Management of the patient	
**Consensus statement 2: Adjunctive therapies**	
Key management options adjunctive to standard pharmacological therapy for AD, VaD, and BPSD include psychosocial interventions, cognitive behavioral therapy, vitamin B, folic acid, and EGb 761^®^	Class of recommendation IIA; level of evidence A
**Consensus statement 3: Management of comorbidities**	
Concomitant management of co‐morbidities, such as hypertension, in patients with AD, VaD, and BPSD is highly important	Expert recommendation

Prevention of dementia	
**Consensus statement 4: EGb 761^®^ does not appear to prevent dementia**	
EGb 761^®^ cannot currently be recommended for the prevention of dementia	Class of recommendation III; level of evidence A

Safety of EGb 761^®^	
**Consensus statement 5: EGb 761^®^ is well tolerated**	
Current safety evidence suggests a good tolerability profile with EGb 761^®^ in the treatment of MCI, AD, VaD, and BPSD	Level of evidence A
**Consensus statement 6: No overall increased bleeding risk**	
Based on existing data in AD, VaD, and mixed dementia (including the GINDEM, GOTADAY, and GOT‐IT! studies), and two meta‐analyses, there appears to be no overall added risk of bleeding with EGb 761^®^	Level of evidence A
Further studies are required in certain patient subgroups, including those with a high cerebral microbleed load (>4 microbleeds) in the cortical areas. In such cases, patients should be warned of a possible increased risk of bleeding	Expert recommendation
**Consensus statement 7: No significant interaction with anticoagulants or antiplatelet agents**	
No significant interaction of EGb 761^®^ with concomitant anticoagulants has been demonstrated	Level of evidence B
No significant interaction of EGb 761^®^ with concomitant antiplatelet agents has been demonstrated	Level of evidence A

Positioning of EGb 761^®^ in dementia treatment algorithms	
**Consensus statement 8: Inclusion in Clinical Practice Guidelines**	
EGb 761^®^ may be considered for incorporation into national CPGs as part of the treatment algorithm for AD, VaD, and BPSD The Expert Group suggested that EGb 761^®^ should be recommended in CPGs, prior to widespread use. Hence, the role of EGb 761^®^ should be discussed in the guidelines of respective Asian countries	Class of recommendation IIA; level of evidence A

AChEI, acetylcholinesterase inhibitor; AD, Alzheimer's disease; BPSD, behavioral and psychological symptoms of dementia; CPG, clinical practice guideline; CVD, cerebrovascular disease; MCI, mild cognitive impairment; VaD, vascular dementia.

**Classes of recommendation**

Class I Evidence and/or general agreement that a given treatment or procedure is beneficial, useful, effective (is recommended/is indicated)
Class IIa Weight of evidence/opinion is in favor of usefulness/efficacy (is reasonable to consider)
Class IIb Usefulness/efficacy is less well established by evidence/opinion (may be reasonable to consider)
Class III Evidence or general agreement that the given treatment or procedure is not useful/effective, and in some cases may be harmful (is not recommended)

**Levels of evidence**

- Data derived from multiple randomized, placebo‐controlled clinical trials, or meta‐analyses
- Data derived from a single randomized clinical trial or large nonrandomized studies
- Consensus of opinion of experts and/or case reports, small studies, retrospective studies

**Table 2:** Efficacy of EGb 761^®^ in key randomized, placebo‐controlled trials

Author, year (study title)	Study design	Patient population	N	EGb 761^®^ dosage	Efficacy Outcomes
Napryeyenko (2007)^31^ (GINDEM‐NP study; Ukraine)	Multicenter, randomized, double‐blind, placebo‐controlled trial	Outpatients aged ≥50 years with mild‐to‐moderate dementia (9‐23 on SKT test battery) Probable AD per NINCDS/ADRDA Possible AD per NINCDS/ADRDA with CVD per NINDS/AIREN criteria Probable VaD per NINDS/AIREN criteria No evidence of other brain lesions	400	2 × 120 mg tablets/day for 22 weeks	Mean –3.2‐point improvement in SKT test score with EGb 761^®^ treatment; mean +1.3‐point increase with placebo (4.5‐point difference; *P* < 0.001) Clinically meaningful improvement in 66% of EGb 761^®^ patients (vs 6% of placebo patients) EGb 761^®^ was significantly superior to placebo on all secondary outcome measures, including NPI and ADL scales Comparable outcomes between AD and VaD patients
Ihl (2011)^1^, ^29^ (GOTADAY study; Ukraine)	Multicenter, randomized, double‐blind, placebo‐controlled trial	Outpatients aged ≥50 years with mild‐to‐moderate dementia, AD, VaD, or mixed (9‐23 on SKT test battery; ≥5 on NPI) Probable AD per NINCDS/ADRDA Possible AD per NINCDS/ADRDA with CVD per NINDS/AIREN criteria Probable VaD per NINDS/AIREN criteria No evidence of other brain lesions	410	240 mg once daily for 24 weeks	Mean –1.4‐point improvement in SKT, and –3.2‐point improvement on NPI total score with EGb 761^®^. With placebo, mean +0.3 point deterioration on SKT, and no change on NPI (*P* < 0.001 for both comparisons) Clinically meaningful improvement in cognition in 32% of EGb 761^®^ patients (vs 15% of placebo patients [*P* < 0.001]) Significant improvement with EGb 761^®^ vs placebo for all secondary endpoints, including apathy/indifference, sleep/night‐time behavior, irritability/lability, depression/dysphoria, and aberrant motor behavior Significant improvement in caregiver wellbeing
Herrschaft (2012)^30^, ^68^ (GOT‐IT! Study; Belarus, Moldova, Russian Federation)	Multicenter, randomized, double‐blind, placebo‐controlled trial	Outpatients aged ≥50 years with mild‐to‐moderate AD, VaD, or AD with vascular components Probable AD per NINCDS/ADRDA Possible AD per NINCDS/ADRDA with CVD per NINDS/AIREN criteria Probable VaD per NINDS/AIREN criteria No evidence of other brain lesions	410	240 mg once daily for 24 weeks	Mean –2.2‐point improvement in SKT with EGb 761^®^ vs a minimal mean –0.3 point improvement with placebo on the SKT. Mean NPI composite score improved by –4.6 in the EGb 761^®^‐treated group and –2.1 in the placebo group (*P* < 0.001 for both comparisons) Clinically meaningful improvement in SKT in 43% of EGb 761^®^ patients (vs 23% of placebo patients [*P* < 0.001]) Secondary variables favored EGb 761^®^, including ADL and QoL measures Significant improvement in caregiver distress
Schneider (2005)^69^ (USA)	Multicenter, randomized, double‐blind, placebo‐controlled trial	Outpatients aged ≥60 years with uncomplicated AD dementia (mean of 18 points on MMSE and <4 on modified HIS)	513	120 or 240 mg/day for 26 weeks	No significant between‐group differences in cognition. Subgroup with NPS showed significantly improved cognitive performance and global assessment scores vs placebo
LeBars (1997)^27^ (USA)	Multicenter, randomized, double‐blind, placebo‐controlled trial	Outpatients ≥45 years Mild‐to‐moderate AD or VaD (score of 9‐26 on MMSE; 3‐6 on GDS scale)	327	3 × 40 mg tablets daily for 52 weeks	EGb 761^®^ improved cognitive performance vs placebo: 1.4‐point improvement in ADAS‐Cog score vs placebo (*P* = 0.04); 27% vs 14% of patients, respectively, achieved at least a 4‐point improvement in ADAS‐Cog (*P* = 0.005). EGb 761^®^ also improved ADL: 0.14‐point improvement in GERRI score vs placebo (*P* = 0.004); 37% vs 23% of patients, respectively, improved on the GERRI scale (*P* = 0.003)
Kanowski (1996)^42^ (Germany)	Multicenter, randomized, double‐blind, placebo‐controlled trial	Outpatients ≥55 years Mild‐to‐moderate AD dementia or multi‐infarct dementia (VaD) (6‐18 on SKT test)	222	2 × 120 mg tablets daily for 24 weeks	28% vs 10% responders with EGb 761^®^ vs placebo (*P* = 0.005)
Gavrilova (2014)^44^ (GIMCIPlus Study; Russia)	Multicenter, randomized, double‐blind, placebo‐controlled trial	Outpatients ≥55 years with MCI with NPS (high baseline incidence of night‐time disruptive behavior and anxiety syndrome; score of ≥6 on NPI)	160	240 mg/day for 24 weeks	EGb 761^®^ improved NPI composite score vs placebo (mean, –7.0 vs –5.5, respectively; *P* = 0.001); improvement by ≥4 points was achieved by 79% vs 56% of patients (*P* = 0.002) EGb 761^®^ also improved cognitive performance and anxiety vs placebo
Grass‐Kapanke (2011)^46^ (Latvia)	Multicenter, randomized, double‐blind, placebo‐controlled trial	Outpatients aged 45‐65 years with vMCI (intact ADL)	300	240 mg once daily for 12 weeks	Significant improvement in attention with EGb 761^®^; trends in favor of EGb 761^®^ in facial recognition and prospective memory, concentration and perceived physical health. Cognitive benefits were more pronounced in subjects with poorer baseline memory function
Zhao (2012)^45^ (China)	Multicenter, randomized, controlled trial (controls received general health care only)	Outpatients aged 60‐85 years with MCI (memory complaints)	120	3 × 19.2 mg doses per day for 6 months	Significant improvement from baseline in logical memory and picture recognition with EGb 761^®^; no significant improvement with health care alone. Clinical and logical memory tests were significantly improved with EGb 761^®^ vs healthcare alone

AD, Alzheimer's disease; ADAS‐Cog, Alzheimer's Disease Assessment Scale (cognitive); ADL, activities of daily living; ADRDA, Alzheimer's Disease and Related Disorders Association; AIREN, Association Internationale pour la Recherche et l'Enseignement en Neurosciences; CVD, cerebrovascular disease; GAS, global assessment scale; GDS, Global Deterioration Scale; GERRI, Geriatric Evaluation by Relative's Rating Instrument; HIS, Hachinski Ischemic Score; MMSE, Mini‐Mental State Examination; NINCDS, National Institute of Neurological and Communicative Disorders and Stroke; NPI, Neuropsychiatric Inventory; QoL, quality of life; SKT, Syndrom‐Kurztest; VaD, vascular dementia; vMCI, very mild cognitive impairment.

### 3.1 Efficacy of EGb 761^®^ in placebo‐controlled trials

Older placebo‐controlled clinical trials have been heterogeneous in study design, EGb 761^®^ dosage, and effect sizes, but overall have shown modest improvement in cognitive function and ADL.^27^, ^42^, ^43^ However, more recently, three multicenter, randomized, double‐blind, placebo‐controlled trials of comparable design were conducted in patients aged ≥50 years with possible/probable AD or probable VaD: the *GINkgo biloba *special extract in DEMentia with NeuroPsychiatric features (GINDEM‐NP),^31^ Ginkgo One Tablet A DAY (GOTADAY),^1^, ^29^ and Ginkgo Once‐daily Tablet‐International Trial (GOT‐IT!)^30^ studies. In these trials, EGb 761^®^ demonstrated significant improvements in cognitive function, NPS/BPSD, ADL, and QoL measures in patients with mild‐to‐moderate dementia, compared with placebo (Table 1).^29^, ^30^

Randomized, double‐blind, placebo‐controlled trials in patients with MCI have also shown significant improvements in NPS, cognitive performance, memory function, concentration, and anxiety with EGb 761^®^ vs placebo.^44^, ^45^

### 3.2 Efficacy findings in key meta‐analyses

Results of a 2014 meta‐analysis by Gauthier and Schlaefke confirmed the efficacy and tolerability of *Ginkgo biloba *extract EGb 761^®^ in patients with dementia. This was a meta‐analysis across all good quality, placebo‐controlled trials with sample sizes of at least 200 randomized patients. Efficacy was demonstrated in the domains of cognition (*P* = 0.03), ADL (*P* < 0.001), and clinical global impression (*P* = 0.01). A dose‐dependent effect on cognition was demonstrated; intake of daily doses of 240 mg was associated with a larger effect. The beneficial effects of EGb 761^®^ were most pronounced in studies that included patients with clinically significant NPS at baseline.^47^

A 2015 meta‐analysis by Hashiguchi et al^48^ also demonstrated efficacy results (Syndrome Kurztest [SKT] scores) in favor of EGb 761^®^, both in the AD and AD + VaD subgroups.

While most of the earlier studies accepted patients with BPSD, only the later studies required clinically significant BPSD as assessed by the Neuropsychiatric Inventory (NPI). Therefore, a second meta‐analysis by von Gunten et al (2016)^49^ specifically analyzed the trials in patients with clinically significant BPSD. In a pooled analysis, EGb 761^®^ at daily dose of 240 mg was efficacious in outpatients suffering from AD, VaD, and AD + CVD. EGb 761^®^ significantly improved cognition, BPSD, caregiver distress related to BPSD, ADL, and overall condition, relative to placebo (*P* < 0.001 for all analyses). This effect was consistently observed across the AD, VaD and mixed dementia subgroups.^49^

Finally, a new meta‐analysis by Savaskan et al^50^ investigated the effects of EGb 761^®^ on specific BPSD symptoms in patients with AD, VaD, or AD + CVD. The pooled analysis revealed significant superiority of EGb 761^®^ over placebo in total scores and in ten single‐symptom scores. Besides the improvement of symptoms present at baseline, the risk of developing new symptoms was decreased with EGb 761^®^. Caregiver distress was also reduced.^50^

### 3.3 Does EGb 761^®^ prevent progression to dementia?

Two multicenter, randomized, double‐blind, placebo‐controlled trials (the GuidAge^51^ and Gingko Evaluation of Memory [GEM]^52^ studies) evaluated the use of EGb 761^®^ in the prevention of dementia. The GuidAge trial included 2,850 French primary care patients with memory complaints, who were prospectively followed up over five years.^51^ The GEM study (USA) randomized a total of 3069 individuals aged ≥75 years with normal cognition or MCI at baseline, and assessed them every six months, with a median follow‐up of 6.1 years.^52^ Neither study demonstrated a clear preventative effect in initial analyses.^51^, ^52^ However, a post hoc analysis of the GuidAge study demonstrated a possible late effect of EGb 761^®^ when a more appropriate statistical test was applied.^53^ Further studies are warranted.

### 3.4 EGb 761^®^ safety

EGb 761^®^ has been shown to have a positive risk‐benefit profile.^42^, ^47^ Studies have consistently shown no significantly increased overall risk of adverse events (AEs) compared with placebo.^27^, ^29^, ^30^, ^31^, ^44^, ^46^ This finding was confirmed in a recent safety meta‐analysis of 44 trials in a total of over 6000 patients; odds ratios for dizziness and nausea in fact favoured EGb 761^®^ over placebo.^54^ Another meta‐analysis showed a numerically lower rate of dropout for any reason in EGb 761^®^‐treated patients vs those receiving placebo (OR, 0.85; 95% CI, 0.67‐1.08). Only patients receiving higher doses of EGb 761^®^ showed significantly higher dropout rates due to side effects, compared with placebo.^48^

In a comparative trial, combination therapy with EGb 761^®^ plus donepezil resulted in fewer AEs compared with donepezil monotherapy.^55^

Some concern has been raised regarding a possible increased risk of bleeding in patients treated with EGb 761^®^. This issue was raised in the late 1990s due to ginkgolide B, a platelet‐activating factor (PAF)‐antagonist component in *Ginkgo biloba*. EGb 761^®^, with its high content of ginkgolides A, B, C, and J was used in a preclinical study, and side effects were reported, but the concentration of these ginkgolides required to inhibit platelet aggregation was high. Evidence shows that the amount of ginkgolide B in a therapeutic dose of EGb 761^®^ is far below what is necessary to inhibit the aggregation of human platelets.^56^, ^57^

Based on outcomes from randomized trials and two meta‐analyses, there appears to be no evidence of an increased risk of bleeding with EGb 761^®^.^40^, ^54^ Clinical studies of EGb 761^®^ have not demonstrated any significant or clinically important changes in coagulation parameters, bleeding time, or platelet aggregation in doses up to 480 mg per day.^40^, ^58^, ^59^ One randomized, placebo‐controlled crossover study tested 29 different coagulation and bleeding parameters with no evidence to substantiate a causal relationship between EGb 761^®^ and hemorrhagic complications.^60^

At 600 mg/d, which is far beyond the recommended daily dose of 240 mg, EGb 761^®^ inhibited ex vivo PAF‐induced platelet aggregation in healthy volunteers, but there was no relevant inhibition of adenosine diphosphate‐ or adrenaline‐induced aggregation, no inhibition of collagen‐induced aggregation, and no effect on bleeding time.^61^ Further, in healthy young male subjects, co‐administration of EGb 761^®^ (240 mg) with acetylsalicylic acid (ASA; 500 mg) did not inhibit, nor potentiate, the effects of ASA on bleeding time, coagulation variables, or platelet aggregation. The absence of an additive effect of EGb 761^®^ in combination with ASA suggests that co‐administration of the two drugs does not constitute a safety risk.^62^ EGb 761^®^ also does not appear to change the pharmacodynamic and pharmacokinetic parameters of simultaneously administered warfarin.^63^

### 3.5 Current guidelines regarding the use of EGb 761^®^

The Expert Panel is in agreement that there is a pressing need to take into account the evidence supporting EGb 761^®^ from the above key clinical trials^1^, ^27^, ^29^, ^30^, ^31^, ^42^, ^44^, ^46^ and recent meta‐analyses.^47^, ^49^, ^50^

Notably, there are no specific diagnostic and treatment guidelines for AD + CVD in Asia,^9^ representing an unmet need in the region. Respective countries have their own guidelines. Countries approach the issue of “level of evidence” slightly differently, based on the system used to evaluate the data, and based on the local classification of EGb 761^®^ (drug vs supplement).

In the World Federation of Societies of Biological Psychiatry (WFSBP) guidelines for the biological treatment of AD and other dementias,^64^, ^65^ a number of principles are emphasized with regard to the development of recommendations, including detailed study assessment, transparency in the description of data, data‐driven analysis of results, only methodologically covered conclusions, analysis in practice with respect to efficacy and side effects, and cautious recommendations.^65^ Subtype groups were clearly defined using international criteria (National Institute of Neurological and Communicative Disorders and Stroke/Alzheimer's Disease and Related Disorders Association [NINCDS/ADRDA]^66^ and National Institute of Neurological Disorders and Stroke/Association Internationale pour la Recherche et l'Enseignement en Neurosciences [NINDS/AIREN]^67^).

The studies upon which the guidelines were based demonstrated significant positive effects of EGb 761^®^ on cognition, ADL, overall condition, NPS, and QoL. In terms of delay in symptom progression and number needed to treat, the effects of EGb 761^®^ were in the same range as those achieved with AChEIs. EGb 761^®^ had the same strength of evidence as AChEIs and memantine (Grade 3 recommendation; Level B evidence). Only EGb 761^®^ had Level B evidence in improving cognition, behavior and ADL in both AD and VaD patients.^65^ Interestingly, one study (GINDON study) showed a trend in favor of EGb 761^®^ plus donepezil vs either agent alone, with regard to clinically meaningful cognitive functioning and global improvement.^55^ Comparative data also demonstrated an improved safety profile with EGb 761^®^ vs AChEIs and memantine.^65^

### 3.6 Expert consensus/conclusion statements

Herein, we summarize the Expert Panel's agreed consensus statements regarding the use of EGb 761^®^ in the management of AD, VaD, BPSD, and MCI, along with other specific statements on clinically relevant issues (Table 2). Consensus statements were primarily based on results from the key randomized, controlled trials and meta‐analyses discussed above.

Members of the board foresee an important role for EGb 761^®^ as an option in the treatment of AD, VaD, and BPSD. It is acknowledged that other agents comprise the current standard of care, particularly in the first‐line setting; clinical data on the use of these agents fall outside the scope of this work.

Of note, members of the board endorsed the benefits of supplemental approaches in the management of AD, VaD, and BPSD, including psychosocial interventions (eg, counseling, psychotherapy, and cognitive behavioral therapy), and supplementation with vitamin B and folic acid in cases of deficiency.

## 4 CONCLUSION

While EGb 761^®^ has not been demonstrated to prevent progression to dementia,^51^, ^52^ the Asian Clinical Expert Group on Neurocognitive Disorders concur that robust evidence supports the inclusion of *Ginkgo biloba *extract EGb 761^®^ 240 mg/d as part of the treatment armamentarium for AD, VaD, BPSD, and MCI. The symptomatic efficacy of this dose of EGb 761^®^ in AD, VaD, and mixed dementia appears comparable with that of AChEIs and memantine, and a lack of efficacy of these standard agents may warrant subsequent challenge with EGb 761^®^. This extract has shown encouraging efficacy in terms of improvement in cognition, behavior, and ability to maintain ADL in patients with AD and also in those with VaD,^65^ as well as reducing caregiver burden.^29^, ^30^

A number of unanswered questions remain regarding the efficacy of EGb 761^®^, and there is a pressing need for further data from well‐designed studies. First, there are many different causes of cognitive impairment,^2^ and no studies have been undertaken to date to establish the efficacy of EGb 761^®^ in different pathophysiological subtypes. Second, apart from the GINDON study^55^ in which showed evidence of improved efficacy with donepezil plus EGb 761^®^ vs either agent as monotherapy, there is currently little evidence supporting the combination of EGb 761^®^ with other agents. More data are needed to evaluate the role of EGb 761^®^ as an add‐on therapy to standard AChEI therapy or memantine. These agents have different modes of action, and the Expert Group feels there may be a future role for combination therapy. There is also a need to evaluate the efficacy of EGb 761^®^ in patients with MCI and AD having small vessel CVD.

With regard to tolerability, safety data for EGb 761^®^ indicate that this agent may prove to be better tolerated among fragile elderly patients than current standard‐of‐care agents.^27^, ^29^, ^30^, ^31^, ^44^ Given its excellent safety profile, patients unable to tolerate side effects from AChEIs or memantine may be suitable candidates for EGb 761^®^ treatment.

Importantly, EGb 761^®^ does not appear to increase overall bleeding risk,^40^, ^58^, ^59^ nor to interact with common antiplatelet agents or anticoagulants.^62^, ^63^ However, these studies were performed in young, healthy volunteers, and it is unclear how accurately the data can be extrapolated to the elderly patient population with multiple co‐morbidities. Further data are also required to address this concern in certain patient subgroups, such as those harboring a high microbleed load; or those with a history of gastrointestinal bleeding or mild renal insufficiency who are usually excluded from clinical trials. In addition, data are needed to assess bleeding risks at higher EGb 761^®^ doses. It is suggested that future studies should include imaging criteria as outcome measures, allowing for better insights into the mechanism of action of EGB 761^®^. The inclusion of gradient recalled echo imaging will also allow for risk estimates of cerebral bleeding among patients with cognitive impairment having microbleeds.

Treatment algorithms for dementia vary between countries, depending on approval, funding constraints, and usual clinical practice. It is recommended that physicians in each country follow their respective local clinical practice guidelines. EGb 761^®^ is currently listed in local clinical guidelines in Germany, Switzerland, and some Asian countries including Indonesia, the Philippines, and China. Asian countries where EGb 761^®^ is *not* listed in local guidelines include India, Malaysia, Singapore, Thailand, and Vietnam. Taken together, the evidence indicates that EGb 761^®^ has a favorable therapeutic index, and it is anticipated that the data and recommendations discussed herein may encourage consideration for the incorporation of EGb 761^®^ into various national guidelines around Asia, to further enhance the symptomatic care of individuals with AD and VaD.

## AUTHOR CONTRIBUTIONS

All authors participated in the Expert Panel from which this document was drafted, and critically reviewed and approved the manuscript. Other than participating in the Expert Panel, Drs Yuda Turana, Kaysar Mamun, Christopher Chen, Reshma Merchant, Jacqueline C Dominguez, Anam Ong, Van Thong Nguyen, Orapitchaya Krairit, Ramesh Thukral, and Yong Ji declare no conflict of interest. Nagaendran Kandiah has received honoraria and CME sponsorship from Lundbeck, Novartis, Pfizer, Schwabe, and Eisai. He has also received research funding from Singhealth Foundation, Media Development Authority of Singapore, National Medical Research Council of Singapore and Ministry of Education, Singapore. Chan Yee Fai has received honoraria and CME sponsorship from Astra Zeneca, DCH Auriga, Eisai, Eli Lilly, Janssen (Johnson & Johnson), Lundbeck, Menarini, MSD, Novartis, and Servier. He has received a Research Grant and Human Capital Development Grant from the Ministry of Health Malaysia, as well as having involvement in Industry Sponsored Research initiated by Janssen (Johnson & Johnson). Yong Fee Mann has received honoraria and CME sponsorship from Schwabe and Eisai, and CME sponsorship from Lundbeck and Norvartis. Suraya Yusoff has received honoraria and CME sponsorship from Lundbeck, Novartis, Pfizer, Schwabe, Sanofi, and Eisai. Encarnita Ampil has received lecture fees from Eisai, Lundbeck, Menarini, Novartis, and Torrent Pharma. Simeon Marasigan has received honoraria for lectures from Eisai, Medichem, Torrent Pharma, E*Chines, and Kusum. Ng Li‐Ling has received honoraria and CME sponsorship from Lundbeck, Novartis, Schwabe, and Eisai. Ralf Ihl received grants/research support or was involved as a consultant, speaker, or in advisory boards, or received author honoraria within the last three years from Alexian Krefeld GmbH, APK, Austroplant, Beltz Test, Bezirksregierung Ansbach, BMFSFJ, BMG, BOD, DNP, EAGP, EU, Friedrichvelag, Hirnliga, Hogrefe, Klinikum Christophsbad Göppingen, Land NRW, Landgericht Aachen, Landgericht Duisburg, Landgericht Düsseldorf, Qualworld, Schwabe, Springer, St. Alexius Krankenhaus Neuss, Stiftung Wohlfahrtspflege, Urban & Vogel, VG Wort, and Wissenschaftliche Verlagsgesellschaft. Vorapun Senanarong has received CME sponsorship from A. Menarini (Thailand) Limited. Chuthamanee Suthisisang has received CME sponsorship from A. Menarini (Thailand) Limited.
